# ﻿*Sedumsimingshanense* (Crassulaceae), a new species from Zhejiang, East China

**DOI:** 10.3897/phytokeys.251.125595

**Published:** 2025-01-10

**Authors:** Shi-Qi She, Yang Zhang, Xin Zhou, Ya-Jun Peng, Shen-Hao Yao, Xing-Xing Zhao, Jia Yang, Yue-Liang Xu

**Affiliations:** 1 Zhejiang Museum of Natural History, Zhejiang, Hangzhou, 310014, China Zhejiang Museum of Natural History Hangzhou China

**Keywords:** ITS, morphological characters, new species, phylogenetic analysis, *Sedumsimingshanense*

## Abstract

In this paper, *Sedumsimingshanense***sp. nov.** is described as a new species based on morphological and molecular analyses, and its taxonomic relationships are discussed. Morphological analysis indicates *S.simingshanense* should be classified in the genus Sedumsect.Sedum and is distinct from the related species *S.xunvense* and *S.formosanum* in the morphology of its solitary, light green and smooth stems, flattened leaves, larger, obovate and spurless sepals, yellow anthers, 22–30 ovules per carpel, oblique follicles, and its habitat on shaded slopes or rocks. Phylogenetic analysis of sequences of the nuclear ribosomal internal transcribed spacer (ITS) also demonstrates that *S.simingshanense* has a highest similarity of only 97.22% with any known species and *S.formosanum* is the closest extant relative of the new species.

## ﻿Introduction

*Sedum*Linnaeus (1753: 430) is the largest genus in the family Crassulaceae with about 470 succulent herbaceous to (sub-)shrubby species (Thiede and Eggli 2007). Species within this genus are widely distributed in the Northern Hemisphere, and are most diverse in the Mediterranean region, Central America, the Himalayas, and East Asia (Stephenson 1994; Thiede and Eggli 2007). *Sedum* can be easily distinguished by its usually alternate leaves, sessile carpels slightly connate at the base, free, mostly yellow or white petals and stamens in two whorls (Thiede and Eggli 2007). However, molecular studies have revealed that *Sedum* is a highly polyphyletic group (Nikulin et al. 2016) which may be due to the high morphological plasticity and variability within the genus (Carrillo-Reyes et al. 2009). In China, 121 species were recorded in the Flora of China (Fu and Ohba 2001). During the past 20 years, about 18 *Sedum* species have been newly described from China (Huang et al. 2023).

During the preparation of the latest edition of the Flora of Zhejiang, a province in southeastern China, botanical expeditions were conducted in Zhejiang and its neighboring regions, resulting in the discovery of a previously unidentified *Sedum* species in June 2023. The species is morphologically similar to species grouping in Sedumsect.Sedum, which is characterized by carpels and follicles adaxially gibbous in the Flora of China (Fu and Ohba 2001). These plants grow on shaded slopes or rocks at an elevation of about 100–650 m in Siming Mountain, Zhejiang.

While these plants have a biennial life form, solitary, robust, light green and smooth fertile stems, flattened leaves, larger, obovate, basally spurless sepals, yellow anthers, 22–30 ovules per carpel, and oblique follicles, they were similar but obviously distinguished from *S.xunvense* (with an annual life form, purple-red and ribbed fertile stems, basally spurred sepals, reddish-brown anthers, and 8–13 ovules) and *S.formosanum* (with a perennial life form, clustered fertile stems, thick and succulent leaves, linear-lanceolate and basally spurred sepals, and erect follicles). In particular, the spurless sepals of these plants were the key differences from both *S.xunvense* and *S.formosanum* which both have spurred sepals.

Living plants were collected and cultivated in Zhejiang Museum of Natural History. After in-depth and careful studies of living materials, we were unable to assign it to any species described so far. The new species keys out as *S.formosanum* in the key in the Flora of China (Fu and Ohba 2001). In order to characterize this putatively new species, we examined its morphological features, and performed molecular phylogenetic analyses in order to reconstruct its relationships with morphologically related species, such as *S.xunvense* and *S.formosanum*. As a result, the different morphological characteristics and geographical distribution, and the separate phylogenetic position of the populations allow us to refer to them as a species new to science, named *Sedumsimingshanense*.

## ﻿Materials and methods

### ﻿Field work and sampling

The specimens were collected in Siming Mountain Geopark (Yuyao county, Zhejiang province) (29°45'46"N, 121°02'02"E, elevation 653.2 m, 9 Jun 2023, Xu 2869), Lingnan Longshan (Shangyu county, Zhejiang province) (29°44'58"N, 121°00'39"E, elevation 472.9 m, 9 Jun 2023, Xu 2867) and Lingnan Fengshuping (Shangyu county) (29°47'25"N, 121°00'49"E, elevation 100.4 m, 9 Jun 2023, Xu 2868). Voucher specimens were deposited at the herbarium of Zhejiang Museum of Natural History (ZM0067398, ZM0067385, ZM0067399). Morphological characters of the new species were examined by digital camera (Nikon, Japan), and were further compared to those of the morphologically similar species in the genus *Sedum*, i.e. *S.xunvense* and *S.formosanum*, with morphological data based on Tang and Huang (1993), Ohba (2001), Fu and Ohba (2001), Choi et al. (2020), Chai et al. (2024) and our own measurements (Table 1).

**Table 1.** Main differences between *S.simingshanense* sp. nov. and the morphologically similar *S.xunvense*, *S.formosanum* and *S.alfredii*.

Characters	*S.simingshanense ^#^*	*S.xunvense*^	S.formosanum*^,$,@^	*S.alfredii ^~^*
Life form	biennial	Annual	perennial	perennial
Habitat	on shaded slopes or rocks	damp ravines, rocks and mossy thickets	rocky crevices along the sea coast	shady moist rocks on forested slopes
Fertile stems	solitary, robust, light green, smooth	solitary, slender, purple-red, ribbed	clustered, robust, green or slightly red, smooth	clustered, purple-red or slightly red, smooth
Leaf blades	flattened, spatulate	flattened, obovate to spatulate	thick and succulent, obovate to spatulate or suborbicular	flattened, obovate, spatulate or linear-cuneate
Leaf size (cm)	1.5–3 × 0.6–0.8	1.3–1.8 × 0.5–0.6	1–1.5 × 0.8–1.2	1.2–3 × 0.2–0.6
Sepals (mm)	obovate, 4–7 × 0.6–4, spurless, free	spatulate to obovate, 2–4 × 0.8–2.8, shortly spurred, free	linear-lanceolate, 2–3 mm long, spurred	linear-spatulate, 3–5 × 1–1.5 mm, spurred
Anther colour	yellow	reddish-brown	yellow	reddish-brown
Ovule number per carpel	22–30	8–13	20–30	(unknown)
Follicles	oblique	Oblique	erect	oblique
Seed shape & size (mm)	oval-ellipsoid, 0.48–0.55 × 0.28–0.30	ellipsoid, 0.6–0.7 × 0.25–0.3	narrowly ellipsoid, 0.4–0.6 × ca. 0.1	ca. 0.6 long

Based on Chai et al. (2024)^, Ohba (2001)*, Tang and Huang (1993)$, Choi et al. (2020)@, Fu and Ohba (2001)~ and this paper (measurements at ZM)#

### ﻿DNA extraction and sequencing

DNA was isolated from freshly collected leaves of the newly discovered species found in Simingshan Geopark and Lingnan Longshan of Shangyu county using the Tiangen plant genomic DNA extraction kit (Tiangen Biotech, Beijing). ITS primers ITS-A (5’-GGAAGGAGAAGTCGTAACAAGG-3’) and ITS-4 (5’-TCCTCCGCTTATTGATATGC-3’) amplifying ITS1, 5.8S rDNA and ITS2 regions were taken from Blattner (1999) and White et al. (1990). PCR program started with 5 min of initial denaturation at 94 °С, followed by 35 cycles of denaturation for 20 sec at 98 °С, annealing for 30 sec at 58 °С Tm for ITS, extension for 45 sec at 68 °С, and final extension for 7 min at 68 °С with KODFX DNA Polymerase (KFX-101, TOYOBO). The PCR products were analyzed using a 1.5% agarose TAE gel and subsequently sequenced by the Beijing Genomics Institute (Shenzhen, China). The newly generated sequences from this study were deposited in the National Center for Biotechnology Information (NCBI) (GenBank accessions: PP464048, PP464049, see Suppl. material 2).

### ﻿Data analysis

To ascertain the phylogenetic position of the newly discovered species, DNA sequences of species in genus *Sedum*, as well as additional outgroup species were used. ITS sequences sourced from the NCBI website (Suppl. material 2) were obtained for the purpose of constructing a phylogenetic tree with Eastern Asian species in the *Acre* clade (Crassulaceae) (Nikulin et al. 2016; Messerschmid et al. 2020; Chai et al. 2024). A total of 76 sequences (including two of *S.simingshanense* and three outgroup species), were selected for subsequent analysis. SeqMan software (Burland 2000) was utilized for the assembly and editing of complementary strands, sequence was aligned with MAFFT v7.505 (Katoh and Standley 2013) using ‘--auto’ strategy and normal alignment mode. Ambiguously aligned fragments were removed using Gblocks 0.91b (Talavera and Castresana 2007) with the following parameter settings: minimum number of sequences for a conserved/flank position (39/39), maximum number of contiguous non-conserved positions (8), minimum length of a block (10), allowed gap positions (with half). ModelFinder v2.2.0 (Kalyaanamoorthy et al. 2017) was used to select the best-fit model using BIC criterion. Best-fit model according to BIC: SYM+I+G4. Maximum likelihood phylogenies were inferred using IQ-TREE v2.2.0 (Nguyen et al. 2015) under the SYM+I+G4 model for 5000 ultrafast (Minh et al. 2013) bootstraps, approximate Bayes test (Anisimova et al. 2011), as well as the Shimodaira–Hasegawa–like approximate likelihood-ratio test (Guindon et al. 2010).

Folding pattern of secondary structure of ITS rRNA: ITS1 and ITS2 region alignment was guided by primary and secondary structure conservation manually (Goertzen et al. 2003; Nikulin et al. 2016). The mfold webserver (Zuker 2003) was used with default conditions to elucidate the folding pattern of secondary structure elements in divergent sequences.

## ﻿Results and discussion

### ﻿Morphological analysis

**Figure 1.**
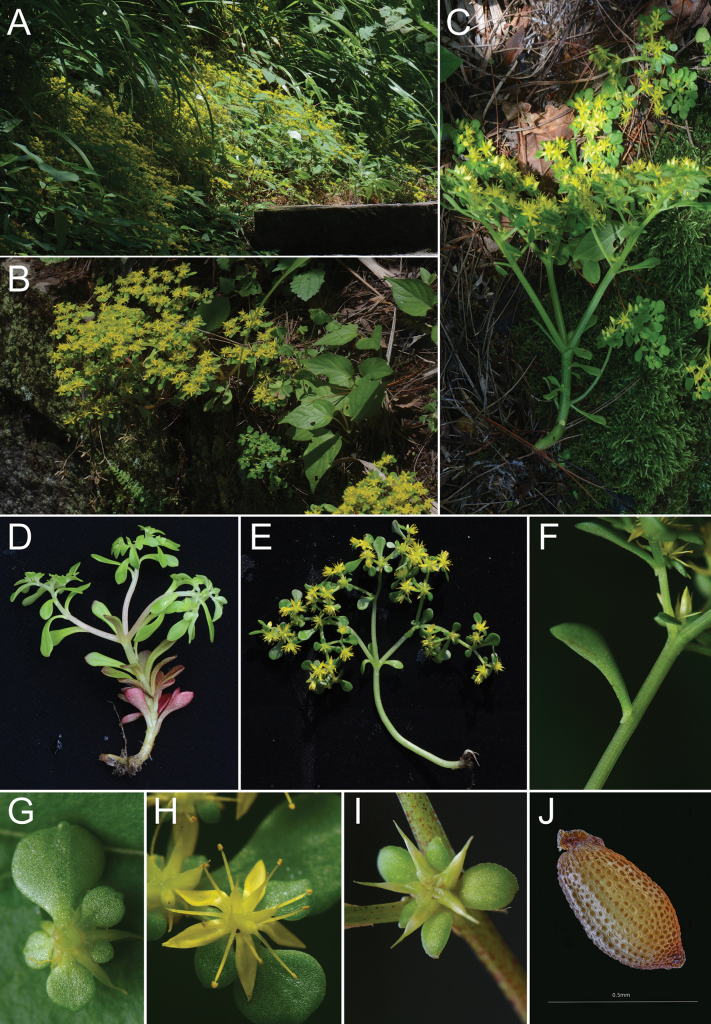
Morphology of *Sedumsimingshanense***A, B** natural habitat under shrubs in deciduous broad-leaved forests (from the type locality in Siming Mountain of Yuyao county, Zhejiang province) **C** flowering specimen **D** specimen before flowering **E** flowering specimen with immature follicles **F** leaf with basal spur **G** sepals from below **H** flower with sepals, petals, stamens and carpels **I** immature follicles **J** unripe seed **A–I** photographed by Yue-Liang Xu in the field (Siming Mountain Geopark, 9 Jun 2023, Xu 2869) **J** photographed by Jian-Sheng Wang in the lab (Lingnan Fengshuping, 9 Jun 2023, Xu 2868).

After field observation, floral morphological characters of the new species were investigated and compared to those of the related species. The new species, named *S.simingshanense*, was found to be clearly different from the other related species (Fig. 1, Table 1). It is mainly distinguished from them by its solitary, light green and smooth stems, flattened leaves, larger, obovate and spurless sepals, yellow anthers, 22–30 ovules per carpel, oblique follicles, and its habitat on shaded slopes or rocks. The distinguishing characteristics of the new species and the three relatives in the *Acre* clade are listed in detail in Table 1.

### ﻿Phylogenetic analysis

**Figure 2.**
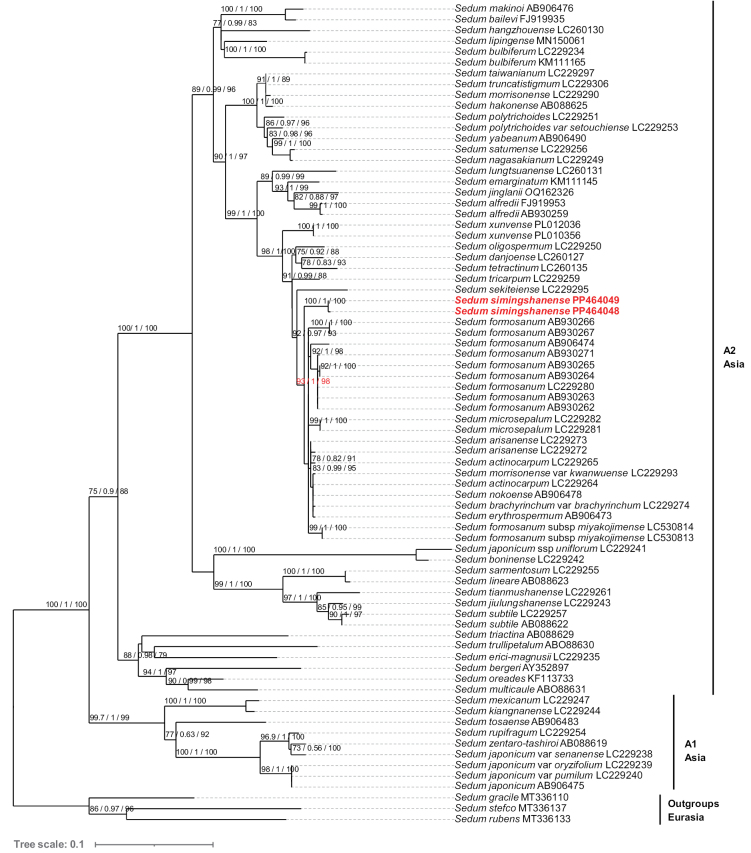
Maximum Likelihood tree based on ITS sequences for Eastern Asian species of the *Acre* clade, and three outgroups of the *Leucosedum* clade (Crassulaceae). Numbers near the branches are ultrafast bootstrap (UFBS) values, Bayesian posteriors (PP) values and SH approximate likelihood ratio test (SH-aLRT) values. UFBS values below 70 were ignored. The new species is highlighted in bold red. The accession numbers from Genbank are indicated after the scientific names. The two major clades in Asian *Sedum* are indicated with A1 and A2.

Our ITS phylogenetic tree presents a topology similar to that of Huang et al. (2023). In addition to the ITS sequences used in the phylogenetic analysis by Huang et al. (2023), 8 ITS sequences most similar to *S.simingshanense*, in descending order, in *S.arisanense* (LC229273), *S.actinocarpum* (LC229264), S.morrisonensevar.kwanwuense (LC229293), S.brachyrinchumvar.brachyrinchum (LC229274), *S.arisanense* (LC229272), *S.danjoense* (LC260127) and *S.xunvense* (PL010356, PL012036) were selected through BLAST searches in the NCBI database (Fig. 2). The phylogenetic analysis shows that the new species *S.simingshanense* is sister to a clade in the *Acre* Clade including *S.formosanum* and eight further *Sedum* taxa, well supported by high ultrafast bootstrap (UFBS) values, Bayesian posteriors (PP) values and SH approximate likelihood ratio test (SH-aLRT) values (93/1/98, Fig. 2) when using Maximum Likelihood method.

According to the predictions, the crassulacean ITS1 and ITS2 are characterized by presence of four helices and five single-stranded areas each (Nikulin et al. 2016). Suppl. material 1 illustrates the proposed base pairing in ITS1 and ITS2 of *S.simingshanense* and *S.formosanum*. Even though related in the phylogenetic tree, *S.simingshanense* and *S.formosanum* have four differences in secondary structures. In ITS1, *S.simingshanense* had an additional loop in Helix I and a side loop (2 nt) changed to an inner loop in Helix III. In ITS2, the two species differed in the structure of Helix I (different position of side and inner loops) and the structure (size) of the terminal loop (3 vs. 6 nt) of Helix II.

## ﻿Discussion

The morphological analysis reveals that while *S.simingshanense*, *S.xunvense* and *S.formosanum* share some similar traits, they exhibit distinct characteristics. In addition to the general features of plants in *S.xunvense*, *S.simingshanense* plants were mainly characterized by its biennial life form (vs. an annual life form), the light green smooth stems (vs. slender, purple-red, ribbed), sepals 4–7 × 0.6–4 mm, basally spurless (vs. 2–4 × 0.8–2.8 mm, basally shortly spurred), yellow anthers (vs. reddish brown), and 22–30 ovules per carpel (vs. 8–13). And in addition to the general features of plants in *S.formosanum*, *S.simingshanense* plants were mainly characterized by its biennial life form (vs. a perennial life form), being solitary (vs. clustered), stems green (vs. green or slightly red), leaves flattened (vs. thick and succulent), sepals obovate, 4–7 × 0.6–4 mm, basally spurless (vs. linear-lanceolate, 2–3 mm long, basally spurred), mature follicles obliquely ascending (vs. erect), and habitat on shaded slopes or rocks (vs. in rock crevices on seashore). In the key for sect. Sedum in the Flora of China (Fu and Ohba 2001), *S.simingshanense* keys out as *S.formosanum*, but differs from that species in its solitary stems while flowering stems of *S.formosanum* are branched from the base.

Furthermore, the molecular findings unambiguously indicate their separate phylogenetic positions. Together, the morphological and molecular evidence substantiate the conclusion that *S.simingshanense* is a distinct new species which groups in the *Acre* clade within the Crassulaceae and in Sedum within the genus *Sedum*.

*Sedumsimingshanense* has only been found in northeastern Zhejiang province of China, whereas *S.xunvense* has only been found in southwestern Zhejiang province (Chai et al. 2024; Fig. 4). *Sedumformosanum* is known to be distributed in the southern region of Japan, the northern islands of the Philippines, Hataedo Island and Sangtaedo Island of Korea, in Taiwan and Fujiang province of China (Rao 1996; Ito et al. 2014a; Choi et al. 2020; Ito et al. 2020). Highlighting the distinctions among these closely related species may provide insights into clarifying the taxonomic relationships within the *Acre* clade of *Sedum*.

### ﻿Taxonomic treatment

#### ﻿ Sedum simingshanense

Taxon classificationPlantaeSaxifragalesCrassulaceae

Y.L. Xu sp. nov.

95C6A5A4-4035-5C3F-A5BE-13018097BE05

urn:lsid:ipni.org:names:77354919-1

Figs 1 , 3

##### Type.

**Figure 3.**
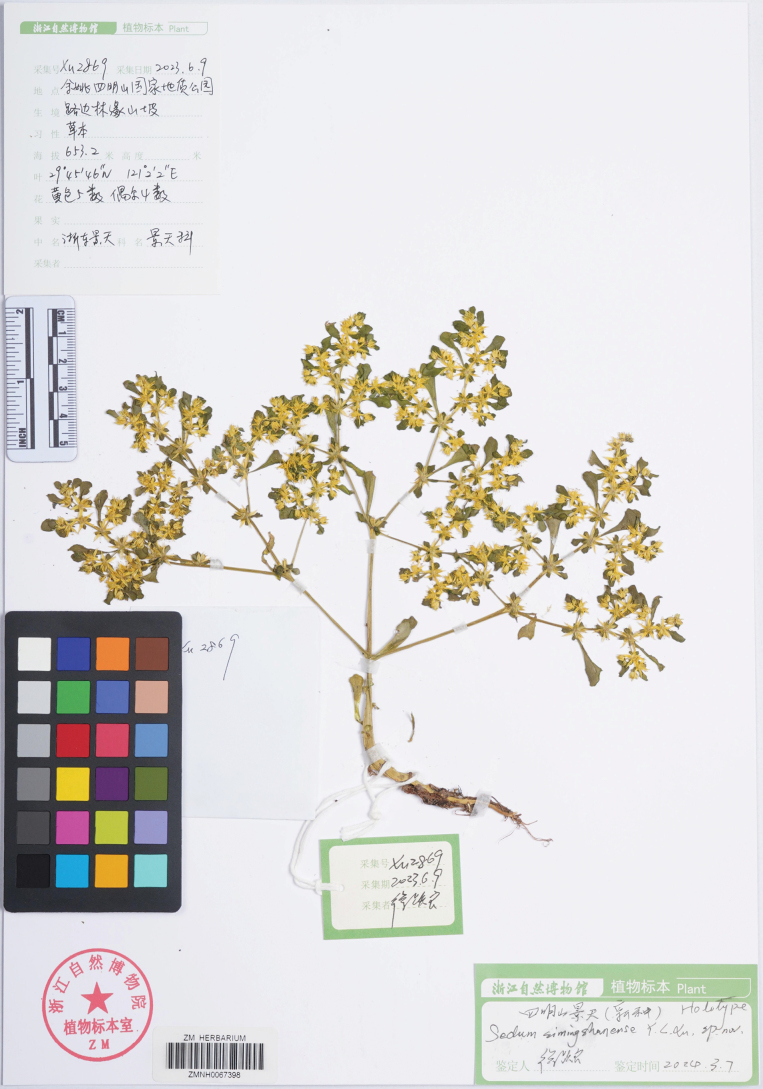
Holotype of *Sedumsimingshanense*. The specimen was collected in Siming Mountain Geopark (Yuyao county, Zhejiang province) (29°45'46"N, 121°02'02"E, elevation 653.2 m, 9 Jun 2023, Xu 2869) (ZMNH0067398).

China • Zhejiang province, Yuyao county, Siming Mountains, Siming Mountain geopark, in roadside slope at forest edge, 29°45'46"N, 121°02'02"E, elevation 653.2 m, 9 Jun 2023, *Yue-Liang Xu*, *Xu 2869* (holotype: ZM barcode ZMNH0067398, Fig. 3).

##### Description.

Biennial herbs, glabrous. Roots fibrous. No sterile stems when flowering. Fertile stems solitary, green, stout and succulent, smooth, branched from above the base into 3 branches, 10–20 cm tall, 5–8 mm in diam. Leaves alternate; leaf blade spatulate, flattened, 1.5–3 cm long, 0.6–0.8 cm wide, apex rounded, base gradually narrowed. Cymes many-flowered, first order branches 3-forked; bracts leaf-like, spatulate to obovate, 0.8–4 cm long, 0.4–1 cm wide, apex rounded, base attenuate into a 0.1–0.15 cm wide pseudopetiole, with short basal spur; flowers usually 5-parted, rarely 4-parted, sepals usually 5, rarely 4, unequal, obovate, thick and fleshy, 4–7 mm long, 0.6–4 mm wide, apex rounded or obtuse, base free, without spur; petals usually 5, rarely 4, yellow, lanceolate, 5–6 mm long, 1.3–2 mm wide, apex acute, base connate for ca. 0.5 mm; stamens usually 10, rarely 8, in 2 whorls, filaments filiform, those opposite to petals with filaments 3–4 mm long, fused with the base of the petal for 1 mm, those opposite to sepals with filaments 4–5 mm long, completely free and not fused; anthers yellow; nectar scales usually 5, rarely 4, pale yellow, oblanceolate, ca. 0.5 mm long, ca. 0.25 mm wide; carpels usually 5, rarely 4, at anthesis upright, in unripe follicles spreading, ovate-lanceolate, 4.5–5 mm long, adaxially gibbous, basally connate for ca. 0.5 mm; styles ca. 0.8–1 mm; ovules 22–30 per carpel. Follicles obliquely diverging. Seeds light brown, oval-ellipsoid, 0.48–0.55 mm long, 0.28–0.30 mm in diam., densely minutely papillate.

##### Distribution and habitat.

**Figure 4.**
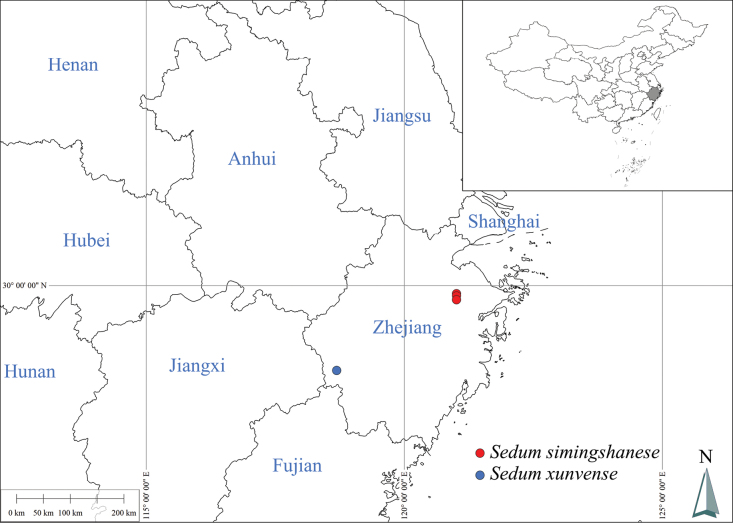
Geographical distribution of *Sedumsimingshanense* (red dots) and *Sedumxunvense* (blue dots) in Zhejiang province of China. The insert map shows the situation of Zhejiang province within China.

The new species is only known from Siming Mountains of Yuyao county, Zhejiang and Lingnan Longshan and Lingnan Fengshuping of Shangyu county, Zhejiang (Fig. 4). It grows on roadside slopes at the forest edge or on moss-covered stone walls at an elevation of about 100–650 m.

##### Phenology.

Flowering from May to June, fruiting in June to July.

##### Etymology.

The specific epithet ‘*simingshanense*’ refers to the type locality of the new species.

##### Similar species.

The new species is similar to *S.xunvense* and *S.formosanum*. It is mainly distinguished from them in the morphology of its solitary, light green and smooth stems, flattened leaves, larger, obovate and basally spurless sepals, yellow anthers, 22–30 ovules per carpel, oblique follicles, and its habitat on shaded slopes or rocks. In the vegetative state, it resembles *Sedumalfredii*, but *S.alfredii* has partly sterile and clustered stems, and purple-red or slightly red and smooth fertile stems (Fu and Ohba 2001). The distinguishing characteristics of the new species and the three morphological relatives are listed in detail in Table 1.

##### Additional specimens examined

**(Paratypes)**. Lingnan Longshan, Shangyu county, roadside slope at forest edge, alt. 472.9 m, 29°44'58"N, 121°00'39"E, 9 Jun 2023, Yue-Liang Xu, Xu 2867 (ZM0067385); Lingnan Fengshuping, Shangyu county, on roadside rocks, alt. 100.4 m, 29°47'25"N, 121°00'49"E, 9 Jun 2023, Yue-Liang Xu, Xu 2868 (ZM0067399).

## Supplementary Material

XML Treatment for Sedum simingshanense

## ﻿Additional information

### Author ORCIDs

Shi-Qi She https://orcid.org/0000-0003-1658-0081

Yang Zhang https://orcid.org/0000-0003-4337-0290

Xin Zhou https://orcid.org/0009-0008-7151-7126

Ya-Jun Peng https://orcid.org/0009-0009-4554-0864

Shen-Hao Yao https://orcid.org/0000-0003-3641-9850

Xing-Xing Zhao https://orcid.org/0009-0003-5808-2758

Jia Yang https://orcid.org/0000-0001-7711-0521

Yue-Liang Xu https://orcid.org/0000-0002-4137-4397

### Data availability

All of the data that support the findings of this study are available in the main text or Supplementary Information.

## Supplementary materials

Supplementary material 1 ITS1 and ITS2 secondary structure analysis of *Sedumsimingshanense* and *Sedumformosanum* based on Mfold predictions Data type tif https://binary.pensoft.net/file/1217368This dataset is made available under the Open Database License (http://opendatacommons.org/licenses/odbl/1.0/). The Open Database License (ODbL) is a license agreement intended to allow users to freely share, modify, and use this Dataset while maintaining this same freedom for others, provided that the original source and author(s) are credited.Shi-Qi She, Yang Zhang, Xin Zhou, Ya-Jun Peng, Shen-Hao Yao, Xing-Xing Zhao, Jia Yang, Yue-Liang Xu

Supplementary material 2 Origin, source and GenBank accession numbers of *Sedum* sequences and three outgroups used for phylogenetic analyses Data type pdf https://binary.pensoft.net/file/1217369This dataset is made available under the Open Database License (http://opendatacommons.org/licenses/odbl/1.0/). The Open Database License (ODbL) is a license agreement intended to allow users to freely share, modify, and use this Dataset while maintaining this same freedom for others, provided that the original source and author(s) are credited.Shi-Qi She, Yang Zhang, Xin Zhou, Ya-Jun Peng, Shen-Hao Yao, Xing-Xing Zhao, Jia Yang, Yue-Liang Xu
