# Supplementary figures and images for: ﻿Sedumsimingshanense (Crassulaceae), a new species from Zhejiang, East China
Source: PhytoKeys. 2025 Jan 10;251:23–35. doi: 10.3897/phytokeys.251.125595 (PMC11742097; doi:10.3897/phytokeys.251.125595)

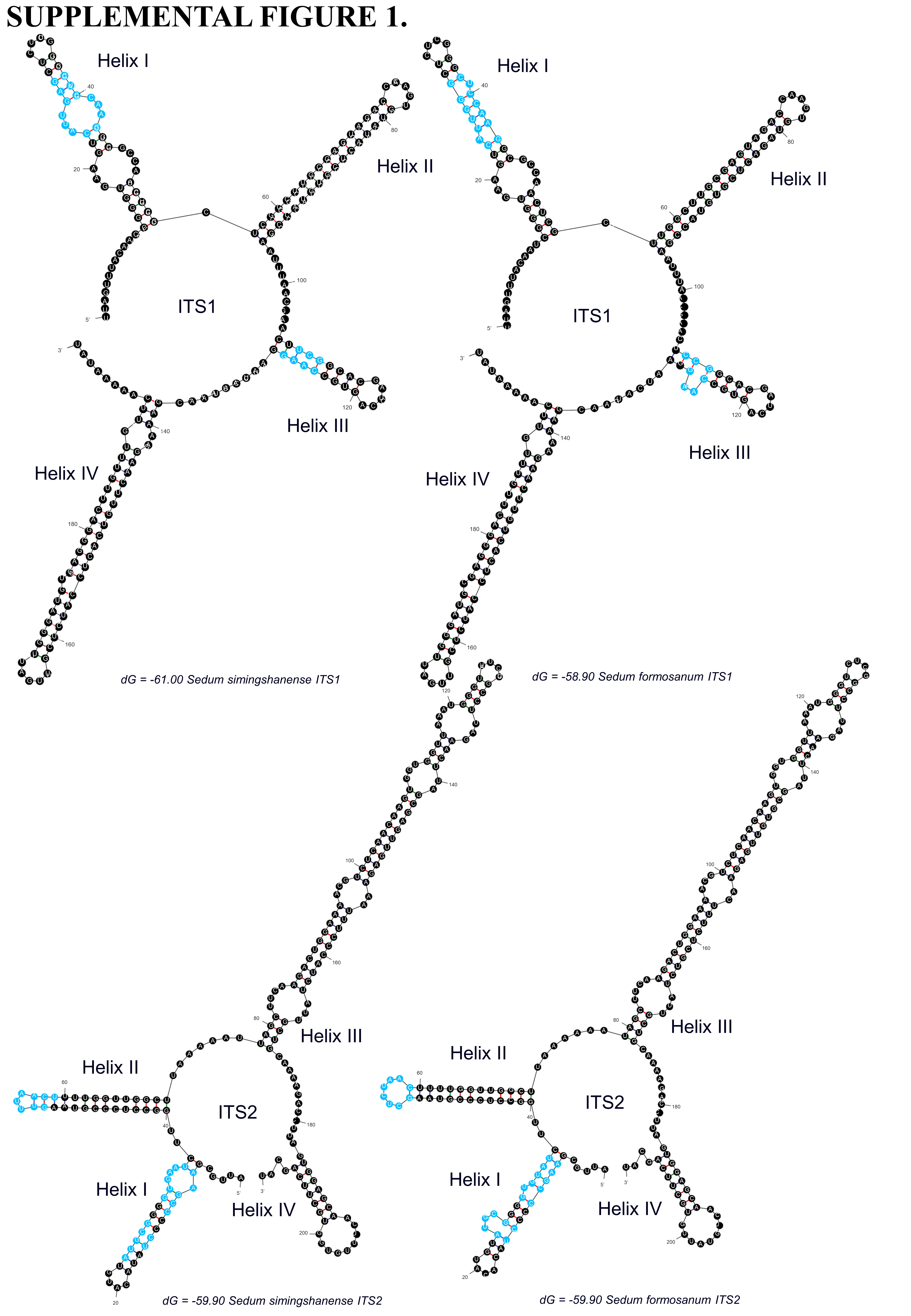

Supplement: Supplementary material 1 — ITS1 and ITS2 secondary structure analysis of Sedumsimingshanense and Sedumformosanum based on Mfold predictions [file phytokeys-251-023_article-125595__-s001.tif]
