# Supplementary material for: ﻿Sedumsimingshanense (Crassulaceae), a new species from Zhejiang, East China
Source: PhytoKeys. 2025 Jan 10;251:23–35. doi: 10.3897/phytokeys.251.125595 (PMC11742097; doi:10.3897/phytokeys.251.125595)
Supplement: Supplementary material 2 — Origin, source and GenBank accession numbers of Sedum sequences and three outgroups used for phylogenetic analyses [file phytokeys-251-023_article-125595__-s002.pdf]

Supplemental TABLE 1. Origin, source and GenBank accession numbers of *Sedum* sequences and three outgroups used for phylogenetic analyses.

Taxon	Voucher	Accession numbers	References
Sedum actinocarpum	Ito 1749	LC229265	Ito et al. 2017b
	Ito 1732	LC229264	Ito et al. 2017b
Sedum alfredi	Kokubugata 17190	AB930259	Ito et al. 2014a
	WUK415208	FJ919953	Wang and Shu unpublished
Sedum arisanense	Ito 1836	LC229272	Ito et al. 2017b
	Ito 1842	LC229273	Ito et al. 2017b
Sedum baileyi	LBG0064555	FJ919935	Wang and Shu unpublished
Sedum bergeri	Ni et al.	AY352897	Ni et al. unpublished
Sedum boninense	Ito 2371	LC229242	Ito et al. 2017b
Sedum brachyrinchum var. *brachyrinchum*	Ito 1359	LC229274	Ito et al. 2017b
Sedum bulbiferum	Ito 416	LC229234	Ito et al. 2017b
	130524qz09	KM111165	Xie et al. 2014
Sedum danjoense	Ito 3658	LC260127	Ito et al. 2017b
Sedum emarginatum	130512hs27	KM111145	Xie et al. 2014
Sedum erici-magnusii	Ito 2077	LC229235	Ito et al. 2017b
Sedum erythrospermum	Tsutsumi 1504	AB906473	Ito et al. 2014b
Sedum formosanum	Kokubugata 11775	AB906474	Ito et al. 2014b
	Kokubugata 16768	AB930262	Ito et al. 2017a
	Kokubugata 16772	AB930263	Ito et al. 2017a
	Kokubugata 16712	AB930264	Ito et al. 2017a
	Kokubugata 15602	AB930265	Ito et al. 2017a
	Kokubugata 12224	AB930266	Ito et al. 2017a
	Kokubugata 10726	AB930267	Ito et al. 2017a
	Kokubugata 6132	AB930271	Ito et al. 2017a
	Ito 1921	LC229280	Ito et al. 2017a

Sedum formosanum subsp. *miyakojimense*	Ito 1115	LC530813	Ito et al. 2020
	Ito 1120	LC530814	Ito et al. 2020
Sedum hakonense	Mayuzumi C00005	AB088625	Mayuzumi and Ohba 2004
Sedum hangzhouense	Ito 2604	LC260130	Ito et al. 2017a
Sedum japonicum	Kokubugata 16749	AB906475	Ito et al. 2014b
Sedum japonicum ssp. *uniflorum*	Ito 447	LC229241	Ito et al. 2017b
Sedum japonicum var. *senanense*	Ito 2200	LC229238	Ito et al. 2017a
Sedum japonicum var. *oryzifolium*	Ito 2285	LC229239	Ito et al. 2017a
Sedum japonicum var. *pumilum*	Ito 2287	LC229240	Ito et al. 2017b
Sedum jinglanii	DNPC 2873	OQ162326	Huang et al. 2023
Sedum jiulungshanense	Ito 76	LC229243	Ito et al. 2017b
Sedum kiangnanense	CMQ1030	LC229244	Ito et al. 2017b
Sedum lineare	Mayuzumi C00120	AB088623	Mayuzumi and Ohba 2004
Sedum lipingense	ZRB1479	MN150061	Zhang et al. 2019
Sedum lungtsuanense	Ito 3563	LC260131	Ito et al. 2017a
Sedum makinoi	Kokubugata 16730	AB906476	Ito et al. 2014b
Sedum mexicanum	Ito 647	LC229247	Ito et al. 2017b
Sedum microsepalum	Ito 1965	LC229281	Ito et al. 2017a
	Ito 2771	LC229282	Ito et al. 2017a
Sedum morrisonense	Ito 2765	LC229290	Ito et al. 2017b
Sedum morrisonense var. *kwanwuense*	Ito 2440	LC229293	Ito et al. 2017b
Sedum multicaule	TI9596136	AB088631	Mayuzumi and Ohba 2004
Sedum nagasakianum	Ito 2064	LC229249	Ito et al. 2017b
Sedum nokoense	Kokubugata 10426	AB906478	Ito et al. 2014b
Sedum oligospermum	Ito 74	LC229250	Ito et al. 2017b
Sedum oreades	Rao 090803-03	KF113733	Zhang et al. 2014
Sedum polytrichoides	CMQ1057	LC229251	Ito et al. 2017b
Sedum polytrichoides var. *setouchiense*	Ito 2298	LC229253	Ito et al. 2017b
Sedum rupifragum	Ito 2070	LC229254	Ito et al. 2017b

Sedum sarmentosum	Ito 978	LC229255	Ito et al. 2017b
Sedum satumense	Ito 2295	LC2292<br>56	Ito et al. 2017b
Sedum sekiteiense	Ito 1456	LC229295	Ito et al. 2017a
Sedum simingshanense	Xu 2869	PP464048	this work
	Xu 2867	PP464049	this work
Sedum subtile	Shimizu 1999	AB088622	Mayuzumi and Ohba 2004
	Ito 2259	LC229257	Ito et al. 2017b
Sedum taiwanianum	Ito 2770	LC229297	Ito et al. 2017b
Sedum tetractinum	Ito 3623	LC260135	Ito et al. 2017a
Sedum tianmushanense	Ito 355	LC229261	Ito et al. 2017b
Sedum tosaense	Kokubugata 16726	AB906483	Ito et al. 2014b
Sedum triactina	9596091	AB088629	Mayuzumi and Ohba 2004
Sedum tricarpum	Ito 2269	LC229259	Ito et al. 2017b
Sedum trullipetalum	Miyamoto et al. 9420132	AB088630	Mayuzumi and Ohba 2004
Sedum truncatistigmum	Ito 3254	LC229306	Ito et al. 2017b
Sedum xunvense	Xu 2864	PL010356	Chai et al. 2024
	Xu 2860	PL012036	Chai et al. 2024
Sedum yabeanum	Ito 396	AB906490	Ito et al. 2014b
Sedum zentaro-tashiroi	Ohba 1998	AB088619	Mayuzumi and Ohba 2004
**outgroup**			
Sedum gracile	ZSS:10 2021 /0	MT336110	Messerschmid et al. 2020
Sedum stefco	ZSS:10 0181 /0	MT336137	Messerschmid et al. 2020
Sedum rubens	E:00764489	MT336133	Messerschmid et al. 2020
